# Assessment of iodine nutrition status in individual adults using machine learning: a cross-sectional study integrating multidimensional features

**DOI:** 10.1186/s12889-026-26420-6

**Published:** 2026-02-06

**Authors:** Zong-Yu Yue, Chun-Hu Li, Ze-Xu Zhang, Meng Zhao, Tong Zhao, Xiang-Kun Zeng, Yu-Hang Liu, Yue Su, Jia Li, Hao-Wen Pan, Xin Hou, Hong-Lei Xie, Peng Liu

**Affiliations:** 1 Center for Endemic Disease Control, Chinese Center for Disease Control and Prevention, Harbin Medical University, No.157, Baojian Road, Nangang District, Harbin, 150081 China; 2 NHC Key Laboratory of Etiology and Epidemiology (Harbin Medical University) & Key Laboratory of Etiology and Epidemiology, Education Bureau of Heilongjiang Province & Heilongjiang Provincial Key Laboratory of Trace Elements and Human Health, Centre for Endemic Disease Control, Chinese Centre for Disease Control and Prevention, Harbin Medical University, Harbin, 150081 China

**Keywords:** Random forest, XGBoost, Iodine nutrition, Forecasting model

## Abstract

**Objective:**

From a public health perspective, the relationship between individual iodine nutritional and its associated risk factors has not been fully elucidated. The aim of this study is to utilize multiple biomarkers to represent individual iodine nutritional status, identify contributing factors for iodine imbalance, and develop a predictive assessment model for iodine nutrition evaluation in different water iodine districts.

**Methods:**

A total of 2,692 participants were recruited from Shandong and Anhui provinces in China. The study population was initially stratified into high-iodine and low-iodine groups based on water iodine concentrations of their residence. Thyroid function indicators and thyroid volume were used as assessment parameters. Both studies first utilized univariate regression to screen variables. After filtering out noisy features, the remaining significant variables were used to split the data into training and testing sets at a 7:3 ratio. Using random forest and eXtreme Gradient Boosting (XGBoost) models, we analyzed how modifiable factors (diet, medical history, lifestyle) relate to iodine homeostasis. Model performance was validated on the testing sets, with accuracy, sensitivity, and area under the curve (AUC) as key metrics.

**Results:**

Integrated analysis of univariate regression, random forest, and XGBoost models revealed significant associations between drinking water sources and disrupted iodine homeostasis. In high-iodine areas, the XGBoost model demonstrated exceptional predictive performance for thyroid volume (R²=0.98, RMSE = 3.53). The results of the random forest classification model showed that the AUC was 0.76 (95% CI: 0.68–0.85) when TSH was used as the assessment indicator, while the AUC for TGAb and TPOAb were 0.74 (95% CI: 0.63–0.84) and 0.67 (95% CI: 0.54–0.80), respectively. In iodine-deficient areas, the XGBoost model maintained good predictive ability for thyroid volume (R^2^ = 0.97, RMSE = 3.28). The random forest model demonstrated moderate diagnostic accuracy among the biomarkers: TSH (AUC = 0.66; 95% CI: 0.51–0.80), TGAb (AUC = 0.69; 95% CI: 0.55–0.83), and TPOAb (AUC = 0.69; 95% CI: 0.56–0.83).

**Conclusion:**

This study established an individualized iodine nutrition assessment model by integrating multi-dimensional biochemical indicators with advanced machine learning algorithms. The model represented by thyroid volume effectively identified the key factors disrupting iodine homeostasis and was capable of accurately predicting individual iodine nutritional status. Its dual utility provides: (1) evidence-based quantitative metrics that can offer personalized guidance for iodine supplementation in clinical practice; and (2) a decision-support framework for regions with varying iodine levels, which can inform the optimization of iodine supplementation programs in specific areas.

**Supplementary Information:**

The online version contains supplementary material available at 10.1186/s12889-026-26420-6.

## Introduction

Iodine is essential for thyroid hormone synthesis and neurodevelopment. Nutritional imbalances can cause severe impairments, particularly in vulnerable populations like neonates [1]. Despite global Universal Salt Iodization (USI) efforts, approximately 30% of the world’s population remains affected by iodine-related disorders [2], while iodine excess has emerged in certain regions [3]. Maintaining optimal iodine homeostasis is critical [4] for individuals but remains challenging due to substantial inter-individual variations in iodine metabolism. While population-level interventions have substantially improved general iodine nutrition, a critical gap remains in the development of validated methods for individualized iodine status assessment.

The assessment of iodine nutritional status currently relies on several key indicators. First, biochemical markers, such as urinary iodine, are widely utilized. Common metrics include median urinary iodine concentration (MUI) and 24-hour excretion. While the MUI reflects total iodine exposure from all sources in a population [5], this metric exhibits temporal variability and is primarily applicable to population-level assessments rather than individual evaluations [6]. Moreover, although 24-hour excretion provides greater individual accuracy, its implementation in large-scale studies is restricted by the logistical burden of sample collection [7]. Second, dietary assessment is frequently employed to estimate intake via questionnaires. However, this method faces significant methodological limitations. Its accuracy is often compromised by inter-individual variability in nutrient absorption rates and unaccounted iodine losses during food storage or cooking processes [8]. Additionally, calculations often depend on average iodine concentrations in food composition databases [9], which may not reflect actual content. Consequently, predictions derived solely from dietary data lack the reliability required for precise iodine nutritional evaluations [10]. Third, clinical biomarkers such as thyroid function parameters (e.g., TSH) are occasionally employed. TSH serves as a monitoring tool for iodine deficiency disorder (IDD) surveillance [11]. However, while TSH is a useful indicator of neonatal status, it demonstrates poor sensitivity in other age groups [12]. Additionally, in patients with thyroiditis or coexisting hypothyroidism, TSH levels respond more sensitively to fluctuations in iodine intake and may be confounded by the presence of thyroid autoantibodies [13]. These limitations render TSH inadequate as a standalone biomarker for comprehensive iodine nutritional assessment.

Thyroid volume (TV), measured via ultrasonography, represents the anatomical size of the gland, with values exceeding established thresholds classified as goiter. As a biomarker, TV is applicable to both population and individual-level assessments [14]. Unlike biochemical indicators, it primarily reflects morphological changes associated with long-term iodine status rather than short-term fluctuations. However, the requirement for specialized equipment and trained personnel restricts its implementation in resource-limited settings. Despite these constraints, TV offers distinct advantages for individual assessment. First, it reflects long-term iodine status and serves as a critical metric for chronic deficiency or excess, playing an irreplaceable role in monitoring [15]. Second, the measurement is non-invasive, universally applicable, and highly reproducible, making it ideal for longitudinal studies. Finally, the intuitive nature of the TV results facilitates patient communication and enhances public awareness of iodine nutrition.

In summary, reliance on a single biomarker is inherently limited by methodological and biological constraints. Iodine status is influenced by multifactorial interactions—spanning dietary habits, environmental exposure, and physiological variability—that traditional statistical models often fail to capture. To address these challenges, a multi-dimensional framework integrating biochemical, dietary, clinical, and morphological indicators is essential for precise individual assessment.

Machine learning (ML) has emerged as a robust tool for automated diagnosis and prognostic evaluation [16]. Univariate regression, effectively used to identify baseline associations and reduce noise, has demonstrated robust stability in nutritional assessments [17]. Additionally, ensemble algorithms like Random Forest and XGBoost facilitate comprehensive multi-factor analysis and feature importance ranking. Collectively, these approaches hold substantial promise for decoding the complex determinants of iodine status, thereby enhancing the precision of individualized assessment [18].

While ML has been successfully applied to predict vitamin deficiencies [19] and evaluate tumor histopathology [20], its utility for individualized iodine assessment remains unexplored. To address this gap, this study integrates multi-dimensional indicators, utilizing univariate regression for preliminary variable screening to remove noise factors. By leveraging the computational power of ensemble algorithms (Random Forest and XGBoost) for automated feature selection and incorporating interpretability frameworks like SHAP values, we developed an individualized iodine nutrition assessment model. Moreover, this study aims to validate the feasibility of using ML to assess individual iodine nutritional status and to provide a scientific basis for developing targeted screening strategies in regions with varying water iodine concentrations.

## Materials and methods

### Study design and sampling methodology

This study utilized survey data from Anhui Province (2018–2022) and Shandong Province (2018), China, to select two regions with distinct iodine exposure levels. A multistage stratified random sampling strategy was employed, leveraging existing data from the National IDD Surveillance and Water Iodine Monitoring Program to identify study sites with varying water iodine concentrations. In Shandong Province, one district or county was randomly selected from each of three distinct water iodine zones. Subsequently, one township was randomly chosen within each selected district or county. To satisfy the predetermined sample size, 1–2 villages were randomly selected per township, and eligible adults were enrolled as participants. The specific study sites included: Dongtan Village and Qianlu Village in Jiaxiang County, Jining City (median water iodine, MWI, < 10 µg/L); Liuxiangzhuang Village and Dongding Village in Weishan County, Jining City (MWI 40–100 µg/L); and Jieyuanji Village in Mudan District, Heze City (MWI > 300 µg/L). All sites were located in southwestern Shandong Province. In Anhui Province, utilizing water iodine data from 2017, site selection was based on historical classifications of high-iodine regions and the status of iodized salt interventions. The sampling sites included: Shaozhuang Village, Huangkou Township, Xiao County, Suzhou City (MWI > 100 µg/L); Liwa Village, Gaoyue Township, Duji County, Huaibei City (MWI > 100 µg/L); Qinglong Village, Qinglong Township, Xiao County, Suzhou City (MWI 40–100 µg/L); and Yuqiao Village, Zhongxing Township, Guzhen County, Bengbu City (MWI < 40 µg/L).

As a cross-sectional study, the expected sample size calculated by the sample size formula (*N = Z*^*2*^_*α/2*_*P (1 - P)/d*^*2*^*(α = 0.05*,*P* = 0.5,* d = 0.05*)) was at least 384 adults in each region. The inclusion criteria for this survey encompassed adults aged 18 to 60 who had resided in the survey area for over five years.

All study protocols were approved by the Ethics Committee of the Endemic Disease Control Center at Harbin Medical University, Chinese Center for Disease Control and Prevention. Written informed consent was obtained from all adult participants prior to enrollment.

### Survey methods and indicators

This cross-sectional study utilized a structured questionnaire administered through face-to-face interviews by trained research assistants. The survey collected data on demographic characteristics, lifestyle factors, comprehensive dietary habits, and relevant health and disease history. To complement the inherent limitations of questionnaire-based iodine status assessment, biochemical analyses were performed to measure serum levels of thyroid-stimulating hormone (TSH), free triiodothyronine (FT3), free thyroxine (FT4), thyroglobulin antibody (TgAb), and thyroid peroxidase antibody (TPOAb). Thyroid morphology was objectively evaluated through standardized ultrasonographic measurement of TV, conducted by certified technicians using a predefined protocol.The comprehensive definitions and specific data collection methods for all study variables are detailed in Supplementary Table 4.

### Evaluation standards

Body mass index (BMI) classifications followed Chinese public health criteria: underweight (BMI < 18.5 kg/m²), normal weight (18.5 ≤ BMI < 24.0 kg/m²), overweight (24.0 ≤ BMI < 28.0 kg/m²), and obesity (BMI ≥ 28.0 kg/m²).

Thyroid function parameters were evaluated based on reference intervals established by clinical laboratory standards. In accordance with the classification recommendations outlined in the Guidelines for the Diagnosis and Management of Thyroid Diseases (Chinese Society of Endocrinology) [21], participants were stratified into “Euthyroid” and “Thyroid Dysfunction” groups to ensure the clinical interpretability of the study results.

The specific diagnostic thresholds were defined as follows: TSH 0.27–4.2 mIU/L, FT4 12–22 pmol/L, FT3 3.1–6.8 pmol/L, TgAb < 115 IU/mL, and TPOAb < 35 IU/mL.

Any indicator deviating from these reference intervals was classified as an abnormality in thyroid function or immune status, serving as the predictive target variable for the ML models.

### Data preprocessing

Following data integration, a rigorous data cleaning process was implemented to mitigate the influence of outliers on the overall dataset. First, surveyed regions were categorized into “Low Water Iodine” and “High Water Iodine” groups based on a water iodine threshold of 100 µg/L, in accordance with the Chinese National Standard (GB/T 19380 − 2016: Definition and demarcation of water-borne high iodine areas) [22]. TV was analyzed as a continuous variable, while thyroid function parameters were treated as categorical variables, with individuals classified according to whether their measurements fell within established normal reference ranges.

To address the class imbalance between the euthyroid and non-euthyroid groups, propensity score matching (PSM) was employed as a robust method for bias reduction and quasi-randomization. This technique balances baseline covariates between groups, thereby minimizing selection bias in comparative analyses [23]. A 1:1 matching of case and control groups was performed based on the following criteria: age ± 5 years, same gender, and BMI ± 3 kg/m^2^. This systematic process established a final balanced cohort for subsequent analyses.

### Statistical analysis

All statistical analyses were performed using SPSS version 25.0 and R software (version 4.3.2), with statistical significance defined as a two-sided *P* < 0.05. Data normality was initially assessed using the Kolmogorov-Smirnov test. Continuous variables with a normal distribution were compared using independent samples t-tests and expressed as mean ± standard deviation (SD). Categorical variables were analyzed using the chi-square test and presented as percentages.

Univariate linear regression was employed for initial feature screening to eliminate noise variables and reduce data dimension. Variables demonstrating statistically significant associations (*P* < 0.05) with the outcome were retained for subsequent analysis. This process refined the feature set, optimizing computational efficiency and focusing on the most informative predictors for subsequent ML model construction.

Given the heterogeneity of iodine nutritional status under different water iodine conditions, Random Forest and XGBoost algorithms were employed to construct stratified models for the high and low water iodine groups, following the exclusion of noise variables via univariate analysis. Given the algorithms’ scale invariance, continuous variables were utilized without normalization, while categorical variables were transformed using one-hot encoding (dummy coding); Multicollinearity was managed intrinsically through node-splitting mechanisms, whereby redundant correlated features are effectively ignored during the greedy selection process. After integrating thyroid function parameters, the dataset was partitioned into training and testing sets at a ratio of 7:3; the same methodology was applied to the TV group. During the model development phase, we explicitly employed a 5-fold cross-validation method combined with a grid search strategy on the training set for hyperparameter optimization. Crucially, feature selection relied on the algorithms’ embedded methods (specifically, Mean Decrease in Gini for Random Forest and Information Gain for XGBoost) and was strictly nested within this cross-validation loop to prevent data leakage and ensure robustness. The final optimized models were validated on the internal testing set (which was not involved in training), where metrics including accuracy, sensitivity, and confusion matrix analysis were calculated to evaluate generalization capability, while SHAP values were computed to quantify feature importance and enhance model interpretability.

## Results

### Basic characteristics of study population

The study enrolled 2,692 participants from Shandong Province (Dongtan Village and Qianlu Village: 409; Liuxiangzhuang and Dongding Village: 392; Yuanyingji Village: 424) and Anhui Province (Shaocun Village: 442, Qinglong Village: 444, Yubei Village: 427, Liwa Village: 427). Participants were stratified into low water-iodine (≤ 100 µg/L) and high water-iodine (> 100 µg/L) groups based on local water iodine concentrations. Iodine nutritional status was assessed using both categorical thyroid function parameters [TSH, FT4, TPOAb, and TgAb] and continuous TV measurements. In the low-iodine group, abnormal values were observed in 113 participants for TSH, 122 for TPOAb, and 126 for TgAb. Corresponding abnormalities in the high-iodine group affected 226 (TSH), 148 (TPOAb), and 177 (TgAb) participants. Additional baseline characteristics are systematically presented in Table 1 and STable 1.

**Table 1:** Table of basic clinical biochemical parameters and anthropometric information

Variables	Low water iodine	High water iodine	*P*
Height (cm)	159.29 ± 7.41	159.41 ± 7.37	0.676
Weight (kg)	65.12 ± 10.97	66.07 ± 11.20	**0.027**
BMI (Kg/m²)	25.62 ± 3.69	25.98 ± 3.88	**0.016**
FT3 (pmol/L)	5.05 ± 1.27	5.42 ± 1.33	0.315
FT4 (pmol/L)	16.47 ± 2.61	16.44 ± 2.99	0.796
TSH (mIU/ml)	2.57 ± 4.78	2.95 ± 3.02	**0.012**
TV (ml)	6.91 ± 4.33	7.06 ± 6.46	0.504
WI (µg/L)	43.93 ± 28.93	193.26 ± 91.23	**< 0.001**
UI (µg/L)	284.25 ± 170.13	482.75 ± 518.37	**< 0.001**
TGAb			**0.018**
No	1151 (90.27)	1220 (87.39)	
Yes	124 (9.73)	176 (12.61)	
TPOAb			0.298
No	1157 (90.75)	1250 (89.54)	
Yes	118 (9.25)	146 (10.46)	
Gender			**0.047**
Female	343 (26.90)	329 (23.57)	
Male	932 (73.10)	1067 (76.43)	

Bold data has statistic significance as their *p*<0.05

### Feature screening using univariate linear regression

The models incorporated thyroid function status (normal/abnormal) and TV as dependent variables. Independent variables included continuous parameters (age, gender, BMI) and categorical covariates (smoking status, alcohol consumption, drinking water source). To ensure the statistical rigor of the model inputs, univariate regression analysis was conducted to identify significant predictors based on a pre-defined significance level of *P* < 0.05.

The preliminary screening outcomes are summarized below.

#### TV

##### Low-iodine group

Variables refined after univariate linear regression screening included gender, smoking status, alcohol consumption, dietary habits, and eight other variables (*N* = 12). All variance inflation factor (VIF) values remained below 10, indicating acceptable multicollinearity. The feature screening is detailed in Stable 2.

##### High-iodine group

Final predictors selected through univariate linear regression comprised gender, smoking status, alcohol consumption, and five other variables (*N* = 8), with all VIF values < 10 (Stable 2).

#### Thyroid function indicators

##### FT4

Owing to the limited sample size, the high- and low-iodine groups were not distinguished when using FT4 as the assessment indicator. The results were as follows: univariate regression screening identified six significant predictors: water source types, kelp consumption, nori intake, dairy product consumption, radiation exposure history, and water iodine levels (Stable 2).

##### TSH

Low-Iodine Group: Four variables demonstrated predictive significance: water source types, nori consumption, smoking status, and UI concentration. High-Iodine Group: Eight retained covariates included water source types, kelp/nori/dairy/fish consumption, cooking oil types, radiation history, and water iodine levels (Stable 2).

##### TPOAb

Low-Iodine Group: Six prognostic factors were retained: egg consumption, dairy intake, saltwater fish consumption, radiation history, UI levels, and body weight. High-Iodine Group: Seven selected variables comprised water source types, kelp/dairy consumption, radiation history, water iodine levels, height, and body weight (Stable 2).

##### TgAb

Low-Iodine Group: Six determinants remained significant: water source types, kelp/egg/dairy/saltwater fish consumption, and UI concentration. High-Iodine Group: Six covariates were retained: water source types, kelp/nori/dairy consumption, radiation history, and water iodine levels (Stable 2).

### XGBoost/ random forest prediction model

#### TV (XGBoost)

**Fig. 1:**
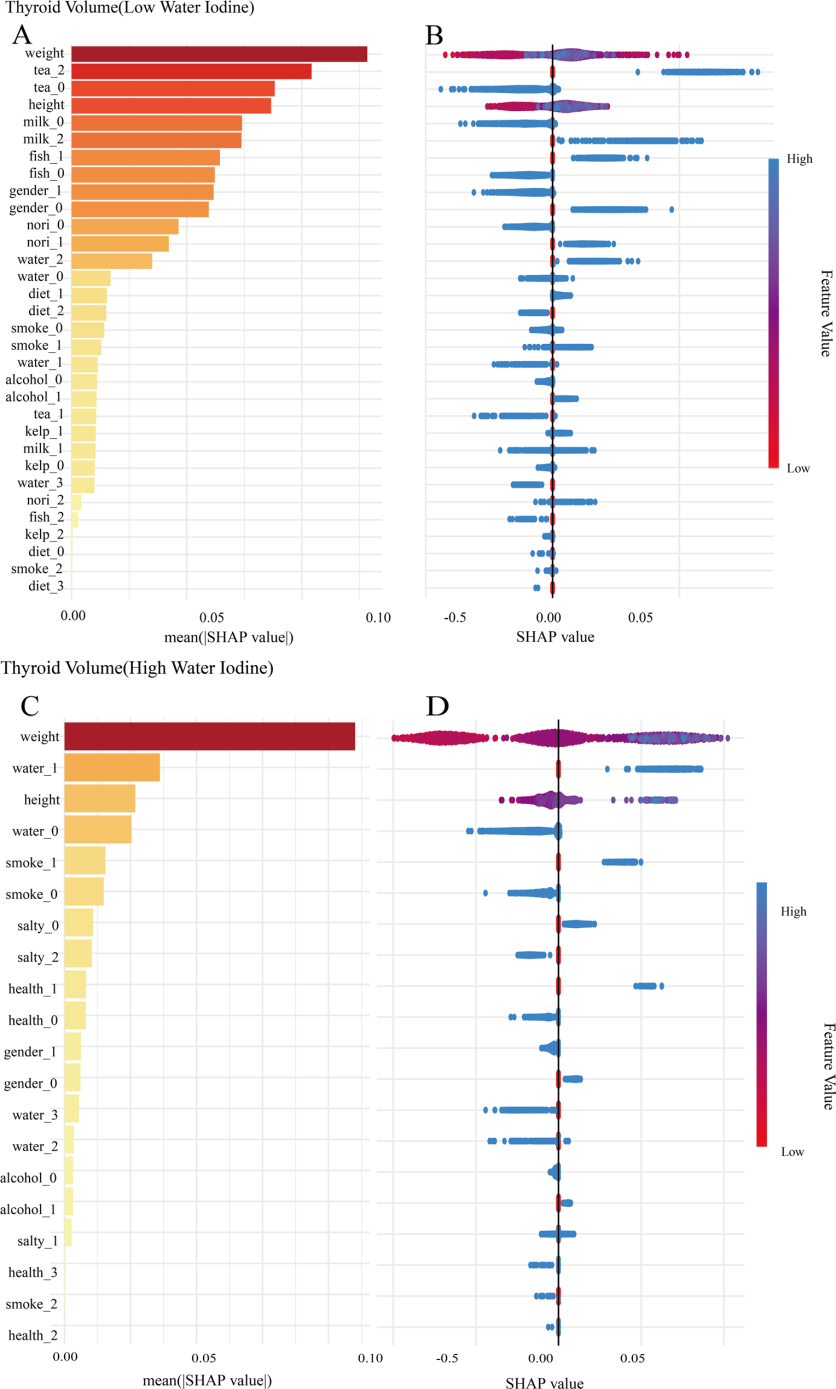
Variable importance distribution plot of thyroid volume in low- and high-water iodine groups. **A** Ranking of importance of thyroid volume variables in low water iodine group. **B** Variable dependence distribution map of thyroid volume in low-water iodine group. **C** Ranking of importance of thyroid volume variables in high-water iodine group. **D** Variable dependence distribution map of thyroid volume in high-water iodine group

**Fig. 2:**
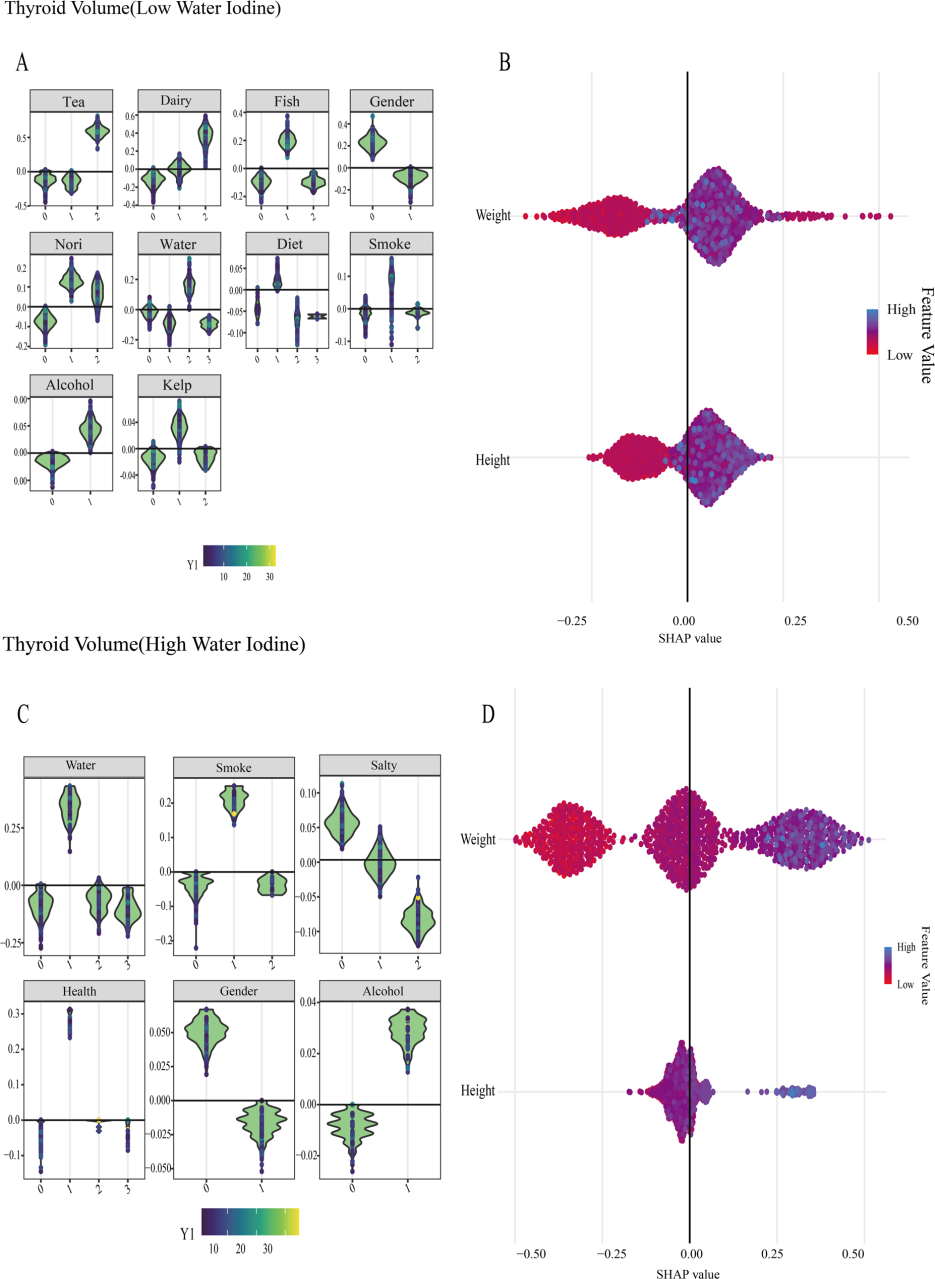
Schematic diagram of thyroid volume group classifications and continuous variable distributions in low- and high-water iodine groups. **A** Schematic diagram of the distribution of thyroid volume categorical variables in the low-water iodine group. **B** Schematic diagram of the distribution of thyroid volume continuous variables in the low-water iodine group. **C** Schematic diagram of the distribution of thyroid volume categorical variables in the high-water iodine group. **D** Schematic diagram of the distribution of thyroid volume continuous variables in the high-water iodine group

In the low-iodine group, SHAP values were utilized to visualize model interpretability. The feature importance ranking demonstrated that body weight exerted the strongest influence on the predictive model. Relationships between variables in the model are illustrated in Fig. 1. Model performance was evaluated using standard regression metrics, and the detailed results for R², MSE, RMSE, and MAE are presented in Table 2. For the high-iodine group, variable importance analysis similarly identified body weight as the most impactful predictor. The inter-variable relationships are presented in Fig. 1. The evaluation metrics for this model are similarly presented in Table 2. The distribution of categorical variables (such as kelp and nori) and continuous variables (such as height and weight) in the model was shown in Fig. 2.

**Table 2:** Predictive performance of machine learning models for key iodine nutritional indicators

Model Target	Iodine Group	Metric Type	*R*² / AUC	MSE/ Accuracy	RMSE/ Sensitivity	MAE / PPV	NPV
TV	Low	Regression	0.967	10.71	3.27	2.27	-
	High		0.986	12.43	3.53	2.45	-
FT4	Combined	Classification	0.763	68.0%	83.0%	58.0%	83.0%
TSH	Low		0.667	73.3%	74.1%	70.2%	76.3%
	High		0.764	67.3%	75.0%	55.9%	79.6%
TPOAb	Low		0.692	60.7%	64.3%	56.3%	65.5%
	High		0.671	56.8%	54.8%	63.9%	50.0%
TGAb	Low		0.691	66.7%	66.7%	55.2%	76.5%
	High		0.739	72.0%	84.6%	63.5%	83.8%

Regression Metrics (for Thyroid Volume): *R*² Coefficient of determination, *MSE *Mean Squared Error, *RMSE *Root Mean Squared Error, *MAE *Mean Absolute Error. Classification Metrics (for Thyroid Function), *AUC *Area Under the Curve, *PPV *Positive Predictive Value, *NPV *Negative Predictive Value. Classification metrics (Accuracy, Sensitivity, PPV, NPV) are presented as percentages (%)

#### Thyroid function indicators (random forest)

##### FT4

**Fig. 3:**
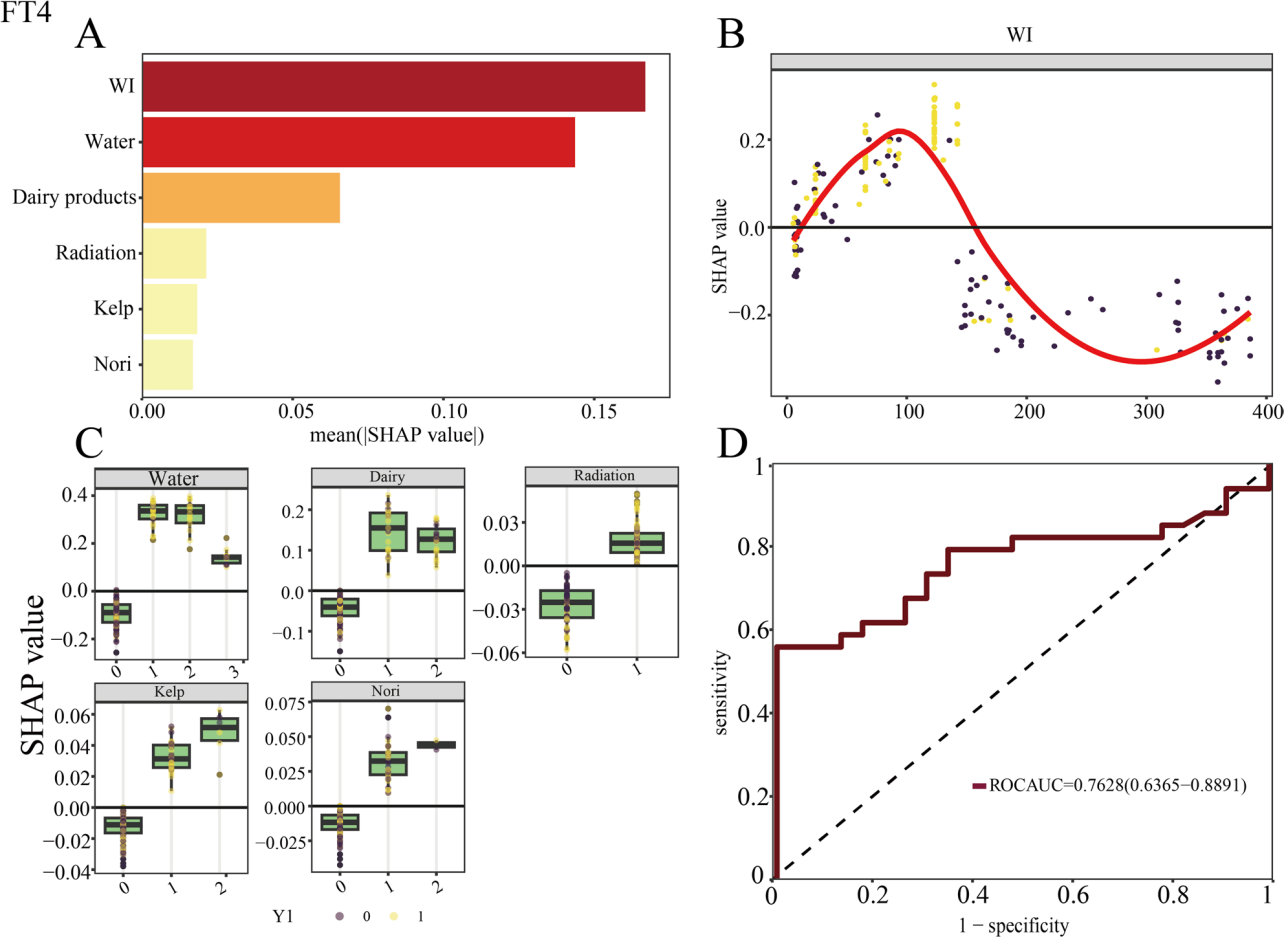
Presentation diagram of the random forest model for FT4 group. **A** Variable Importance Distribution Plot for FT4 Group. **B** Distribution Schematic of the Continuous Variable (Water Iodine Concentration). **C** Distribution Schematic of Categorical Variables. **D** Receiver Operating Characteristic Curve of FT4 Group

In the FT4 group, the optimal mtry parameter for the random forest model was determined to be 3, with model error stabilizing at approximately 30.18% when the number of trees (ntree) reached 414. Subsequently, Fig. 3 illustrates the variable importance ranking derived from the mean decrease in Gini index and SHAP value distributions across predictors. The analysis identified water iodine concentration as the most critical variable in the predictive model. Detailed relationships for categorical and continuous variables are further visualized in Fig. 3.

Model validation included the construction of a receiver operating characteristic (ROC) curve, yielding an area under the curve (AUC) of 0.763 (Fig. 3). Confusion matrix analysis results, including classification accuracy, sensitivity, positive predictive value (PPV), and negative predictive value (NPV), are presented in Table 2.

##### TSH

In the TSH low-iodine group, variable importance analysis identified UI concentration as the most influential predictor. SHAP value distributions for both categorical and continuous variables are presented in Supplementary Fig. 1. The model achieved an area under the ROC curve (AUC) of 0.667. The evaluation results from the confusion matrix analysis, including accuracy, sensitivity, PPV, and NPV are presented in Table 2. For the TSH high-iodine group, variable importance ranking similarly highlighted water iodine concentration as the dominant predictor (Supplementary Fig. 1). SHAP value visualizations specific to categorical variables are further detailed in Supplementary Fig. 1, with the model yielding an AUC of 0.764 (Supplementary Fig. 1). The results of the confusion matrix metrics are presented in Table 2.

##### TPOAb

In the TPOAb Low-Iodine group, variable importance analysis identified UI concentration as the most influential predictor. SHAP value distributions for categorical and continuous variables are presented in Supplementary Fig. 2, with the model achieving an area under the ROC curve (AUC) of 0.692 (Supplementary Fig. 2). For the TPOAb high-iodine group, variable importance ranking highlighted water iodine concentration as the dominant predictor. SHAP value visualizations specific to categorical variables are detailed in Supplementary Fig. 2, and the model yielded an AUC of 0.671 (Supplementary Fig. 2). The confusion matrix results for both the low-iodine and high-iodine groups are presented in Table 2.

##### TGAb

In the TgAb low-iodine group, the analysis identified drinking water type as the most significant variable in the risk assessment model. SHAP value visualizations for categorical and continuous variables are presented in Supplementary Fig. 3, with the model achieving an area under the ROC curve (AUC) of 0.691 (Supplementary Fig. 3). For the TgAb high-iodine group, variable importance analysis highlighted water iodine concentration as the dominant predictor (Supplementary Fig. 3). SHAP value distributions for key model variables are further detailed in Supplementary Fig. 3, and the model yielded an AUC of 0.739 (Supplementary Fig. 3). The confusion matrix results for both the low-iodine and high-iodine groups are presented in Table 2.

## Discussion

By employing Random Forest and XGBoost algorithms, this study successfully developed assessment models for individual iodine nutritional status based on TV and thyroid function parameters, respectively, which outperformed traditional statistical methods. First, traditional statistical methods, such as multiple linear regression and mixed-effects models, possess strong capabilities when handling linear relationship problems [24]. However, this study found a significant non-linear relationship between water iodine concentration and thyroid indicators, indicating that the human physiological response to iodine and individual iodine nutritional status do not follow a simple linear pattern; in contrast, ML algorithms demonstrate superior sensitivity in identifying and modeling these complex non-linear patterns. Furthermore, individual iodine nutritional status is influenced by multiple factors, including diet, environment, and lifestyle [25]. When facing complex interaction terms, traditional models often require manually presetting rules during processing, whereas ML models can implicitly detect the complex non-linear relationships between dependent and independent variables, while simultaneously detecting all possible interactions among predictor variables [26]. In a multi-factorial context, ML methods can provide more stable assessment capabilities.

This study found that, compared with thyroid function indicators, the model based on TV demonstrates higher reliability in evaluating individual iodine nutritional status. This conclusion is supported by three primary considerations. First, the utility of thyroid function indicators, such as TSH, is limited by age-specific factors. As demonstrated by Barona-Vilar et al. [27], while TSH serves as a reliable biomarker for assessing iodine status in neonates, its diagnostic accuracy in adult populations remains questionable. Second, in contrast to TV, thyroid function parameters are prone to rapid physiological fluctuations following acute iodine exposure. Experimental evidence indicates that an acute increase in dietary iodine intake induces a transient surge in TSH levels, which typically returns to baseline within seven days [28]. This short-term reactivity introduces temporal instability, whereas TV reflects chronic iodine status through gradual morphological adaptations, thereby minimizing the impact of transient dietary confounders. Finally, thyroid disorders (e.g., autoimmune thyroiditis, subclinical hyperthyroidism) disproportionately alter thyroid hormone levels, potentially decoupling biochemical parameters from actual iodine status. TV measurements, while not entirely immune to pathological influences, demonstrate greater specificity for nutritional assessment in euthyroid adults.

This study revealed that frequent tea consumption might influence TV through mechanisms beyond variations in water type and iodine content. The observed effects could be attributed to two primary factors. First, tea contains abundant polyphenolic flavonoids, including catechins, which are compounds with demonstrated antithyroid and goitrogenic properties. Animal studies have shown that the administration of black tea extract at a concentration of 5.0% (w/v) induces thyroid enlargement, accompanied by follicular hypertrophy and hyperplasia [29]. Second, fluoride accumulation occurs during tea cultivation and processing. Experimental evidence indicates that fluoride interferes with thyroidal iodine uptake; specifically, cross-sectional research [30] has reported altered thyroid function under low-to-moderate fluoride exposure (50–150 µg/L). Epidemiological data further confirm positive correlations between chronic high-fluoride drinking water consumption (> 1.5 mg/L) and the prevalence of adult goiter or the risk of hypothyroidism [31]. These findings collectively suggest that excessive tea intake may contribute to TV enlargement and the disruption of iodine homeostasis in adults.

This study further demonstrated that both smoking and alcohol consumption exert measurable effects on TV in adults, consistent with previous epidemiological findings. Individuals in iodine-deficient regions tend to exhibit larger TV [32]. Smoking is associated with decreased TSH levels and increased thyroid hormone concentrations [33], potentially due to thiocyanate-mediated competitive inhibition of iodine uptake. Notably, smoking demonstrates enhanced goitrogenic effects in low-iodine areas, where it elevates serum thiocyanate levels and exacerbates iodine utilization deficits [34]. Additionally, benzo pyrene in tobacco smoke stimulates sympathetic nervous activity, thereby interfering with normal thyroid function [35]. Parallel studies have confirmed an association between alcohol consumption and TV enlargement. Epidemiological data revealed a dose-response relationship between alcohol intake levels and TV in both males and females [36]. Alcohol may directly inhibit thyroid function through cytotoxic effects and indirectly suppress thyroid activity by attenuating thyrotropin-releasing hormone responses [37]. Chronic alcohol consumption is further linked to impaired thyroidal iodine uptake, increasing the risk of TV enlargement.

Meanwhile, this study revealed that obesity is associated with an increased TV, which is consistent with the findings of previous epidemiological studies [38]. Previous studies have indicated that obese patients often present with insulin resistance [39], the progression of which may lead to hyperinsulinemia. Notably, Nino Lomtadze et al. [40] demonstrated that patients with insulin resistance exhibit a significantly elevated mean TV, while hyperinsulinemia shows a significant positive correlation with goiter development.

In terms of diet, this study demonstrated that sea product consumption (including kelp, nori, and marine fish) increased the risk of iodine nutritional imbalance, regardless of whether TV or thyroid function parameters were used as the gold standard. This phenomenon might be attributed to the following factors.

First, the iodine content of these foods plays a critical role. Kelp, a marine algae, contains exceptionally high iodine levels-approximately 2,500–3,000 µg per 100 g of fresh kelp, with even higher concentrations observed in dried kelp. Frequent intake, even in iodine-deficient regions, may lead to TV enlargement and functional dysregulation due to excessive iodine exposure. However, given that the intake of kelp remains infrequent and the portions are small across most geographical areas, reaching a level of iodine excess through typical dietary patterns remains uncommon. Additionally, seaweed and marine fish can bioaccumulate heavy metals (e.g., arsenic and lead) from marine ecosystems. These contaminants can inhibit thyroid peroxidase (TPO) activity and disrupt thyroid hormone synthesis. John D. Meeker et al. [41] reported that lead and copper exposure correlate with non-monotonic reductions in TSH levels, while arsenic interferes with enzymes involved in thyroid hormone synthesis and signaling, exhibiting a dose-dependent association with elevated TSH. While seaweed consumption plays a critical role in maintaining iodine homeostasis, indiscriminate intake without regard to frequency or dose may destabilize individual iodine equilibrium.

Excessive consumption of dairy products in low-water-iodine areas may aggravate iodine nutritional imbalance, potentially attributable to several mechanisms. First, variability in iodine content across different dairy products (e.g., milk, cheese, and yogurt) plays a critical role. During processing, curd separation leads to significant iodine loss due to its water-soluble nature [42]. Plant-based dairy alternatives (e.g., soy milk) further compound this issue, as their inherently low iodine content fails to meet dietary requirements in iodine-insufficient regions [43]. Second, bioactive compounds in dairy products may disrupt iodine utilization. Soy-based products contain high levels of isoflavones-nonsteroidal compounds reported to inhibit TPO activity [44]. TPO suppression directly impairs thyroid hormone synthesis, thereby reducing iodine metabolic efficiency and contributing to dysregulated iodine homeostasis [45]. Finally, additives introduced during processing or storage may interfere with iodine absorption. For instance, thiocyanate, a preservative widely used in dairy packaging, exerts potent antithyroid effects. Epidemiological studies have demonstrated an association between thiocyanate exposure and elevated TSH levels [46].

When using thyroid function parameters as the gold standard for assessment, this study found that drinking water sources significantly influenced thyroid function in adults, consistent with prior research.

First, household water serves as a primary iodine source for certain populations [47]. In low-water-iodine regions, chronically low iodine intake from water compromises thyroid hormone synthesis, leading to abnormally elevated TSH levels. Conversely, in high-water-iodine regions, TSH elevation may reflect individual susceptibility. Evidence suggests that most individuals transiently exhibit the Wolff-Chaikoff effect under iodine excess—a protective mechanism where elevated intrathyroidal iodine suppresses iodine organification and transiently reduces hormone synthesis, followed by homeostatic recovery [48]. However, vulnerable subpopulations (e.g., individuals with autoimmune thyroid diseases) may fail to adapt, resulting in sustained suppression of hormone synthesis and persistent TSH dysregulation. Critically, excessive iodine intake amplifies the risk of subclinical hypothyroidism, further elevating TSH levels. Furthermore, rural groundwater contamination (e.g., nitrate pollution) may compound these effects. Specifically, nitrates competitively inhibit iodine uptake via the sodium-iodide symporter (NIS) [49], exacerbating iodine deficiency in low-iodine contexts and synergistically elevating TSH levels. Collectively, these mechanisms disrupt iodine homeostasis, highlighting the necessity for region-specific water iodine surveillance and tailored interventions.

### Strengths and limitations

Through methodological refinements, this study advanced the assessment of iodine nutrition. Unlike previous studies that relied solely on logistic regression, we developed an individualized evaluation model using ML algorithms (Random Forest and XGBoost), which excelled in capturing nonlinear relationships, high-dimensional feature interactions, and complex dependencies inherent in nutritional datasets. The interpretability of the model was enhanced by employing SHAP values and feature importance rankings, allowing for the identification of key predictors such as dietary patterns. Furthermore, our multimodal approach integrated both thyroid function parameters (e.g., TSH and FT4) and TV measurements, providing a dual perspective on iodine status that addressed the limitations of single-metric evaluations.

However, several limitations warrant consideration. The sample size of some individuals with thyroid dysfunction was limited and may be subject to selection bias; therefore, future studies need to expand the sample size. Additionally, the absence of external validation restricted the generalizability of the model, necessitating cross-regional studies with diverse populations to confirm its real-world applicability.

## Conclusion

This study conducted a cross-sectional survey of adults in Shandong and Anhui provinces in China, establishing an assessment model using statistical methods such as Random Forest and XGBoost to screen factors associated with individual iodine nutrition in this population. The model achieved favorable results, demonstrating significant predictive capability. Concurrently, key variables contributing to increased risks of individual iodine nutritional imbalance were identified, and corresponding mitigation strategies were proposed. To ensure iodine nutritional homeostasis in adults, regions should tailor resident’s iodine intake based on local conditions. Simultaneously, adults should modify unhealthy habits according to personal circumstances and implement multi-level and multi-faceted prevention and intervention strategies to improve their overall health status.

## Supplementary Information

Supplementary Material 1.

## Data Availability

The datasets analysed in this study are not publicly available due to patient confidentiality, but are available from the corresponding author on reasonable request.

## Abbreviations

AFRs: Alternative flame retardants
BMI: Body mass index
FT3: Free Triiodothyronine
FT4: Free Thyroxine
MUI: Median urinary iodine concentration
ML: Machine learning
NPV: Negative predictive value
PPV: Positive predictive value
TV: Thyroid volume
TSH: Thyroid stimulating hormone
TgAb: Thyroglobulin antibodies
TPOAb: Thyroid peroxidase antibodies
UI: Urinary Iodine
UIC: Urinary iodine concentration
USI: Universal salt iodization
MWI: Median water iodine
XGBoost: eXtreme Gradient Boosting
