# Supplementary material for: Assessment of iodine nutrition status in individual adults using machine learning: a cross-sectional study integrating multidimensional features
Source: BMC Public Health. 2026 Feb 6;26:843. doi: 10.1186/s12889-026-26420-6 (PMC12977894; doi:10.1186/s12889-026-26420-6)
Supplement: Supplementary file 1 — Supplementary Material 1. [file 12889_2026_26420_MOESM1_ESM.docx]

Stable 1 Lifestyle and Dietary Habits Questionnaire for Study Participants

| Variables | Low water iodine | High water iodine | *P* |
| --- | --- | --- | --- |
| **Smoke** |  |  | 0.505 |
| Never smoker | 1047 (82.12) | 1172 (83.95) |  |
| Current smoker | 207 (16.24) | 203 (14.54) |  |
| Former smoker | 21 (1.65) | 21 (1.50) |  |
| **Alcohol** |  |  | 0.191 |
| No | 958 (75.14) | 1079 (77.29) |  |
| Yes | 317 (24.86) | 317 (22.71) |  |
| **Tea** |  |  | 0.680 |
| Rarely | 969 (76.00) | 1065 (76.29) |  |
| Drink occasionally | 83 (6.51) | 100 (7.16) |  |
| Drink often | 223 (17.49) | 231 (16.55) |  |
| **Water** |  |  | **<0.001** |
| Tap water | 724 (56.78) | 798 (57.16) |  |
| Centralized well water | 151 (11.84) | 291 (20.85) |  |
| Private well water | 271 (21.25) | 146 (10.46) |  |
| Bottled water | 129 (10.12) | 161 (11.53) |  |
| **Eating habits** |  |  | 0.191 |
| Strictly vegetarian | 19 (1.49) | 28 (2.01) |  |
| Predominantly vegetarian | 993 (77.88) | 1114 (79.80) |  |
| Predominantly meat-based | 261 (20.47) | 249 (17.84) |  |
| Strictly meat-based | 2 (0.16) | 5 (0.36) |  |
| **Saltiness Preference** |  |  | 0.148 |
| Salty | 515 (40.39) | 539 (38.61) |  |
| Moderate | 431 (33.80) | 522 (37.39) |  |
| Bland | 329 (25.80) | 335 (24.00) |  |
| **Kelp** |  |  | 0.451 |
| Rarely | 793 (62.20) | 877 (62.82) |  |
| Consume occasionally | 421 (33.02) | 466 (33.38) |  |
| Consume regularly | 61 (4.78) | 53 (3.80) |  |
| **Nori** |  |  | 0.114 |
| Rarely | 781 (61.25) | 882 (63.18) |  |
| Consume occasionally | 417 (32.71) | 454 (32.52) |  |
| Consume regularly | 77 (6.04) | 60 (4.30) |  |
| **Egg** |  |  | 0.183 |
| Rarely | 90 (7.06) | 122 (8.74) |  |
| Consume occasionally | 203 (15.92) | 201 (14.40) |  |
| Consume regularly | 982 (77.02) | 1073 (76.86) |  |
| **Dairy products** |  |  | **0.007** |
| Rarely | 758 (59.45) | 891 (63.83) |  |
| Consume occasionally | 253 (19.84) | 214 (15.33) |  |
| Consume regularly | 264 (20.71) | 291 (20.85) |  |
| **Fish** |  |  | **0.023** |
| Rarely | 830 (65.10) | 978 (70.06) |  |
| Consume occasionally | 408 (32.00) | 382 (27.36) |  |
| Consume regularly | 37 (2.90) | 36 (2.58) |  |
| **Types of edible oil** |  |  | **0.017** |
| Vegetable oil | 1163 (91.22) | 1278 (91.55) |  |
| Animal fat | 6 (0.47) | 3 (0.21) |  |
| Both | 53 (4.16) | 35 (2.51) |  |
| Others | 53 (4.16) | 80 (5.73) |  |
| **Dietary Supplements** |  |  | 0.635 |
| None | 1171 (91.84) | 1267 (90.76) |  |
| Minerals | 55 (4.31) | 67 (4.80) |  |
| Multivitamins | 11 (0.86) | 10 (0.72) |  |
| Others | 38 (2.98) | 52 (3.72) |  |
| **Tumor Conditions** |  |  | **0.033** |
| No | 1268 (99.45) | 1377 (98.64) |  |
| Yes | 7 (0.55) | 19 (1.36) |  |
| **Timing of Salt Addition** |  |  | 0.188 |
| At the start of cooking | 177 (13.88) | 157 (11.25) |  |
| During cooking | 830 (65.10) | 922 (66.05) |  |
| After cooking | 242 (18.98) | 289 (20.70) |  |
| Randomly added | 26 (2.04) | 28 (2.01) |  |
| **Cardiovascular Disease** |  |  | 0.651 |
| No | 1131 (88.71) | 1246 (89.26) |  |
| Yes | 144 (11.29) | 150 (10.74) |  |
| **History of Neck Radiation** |  |  | 0.887 |
| No | 912 (71.53) | 1002 (71.78) |  |
| Yes | 363 (28.47) | 394 (28.22) |  |

Stable 2 Univariate regression variable selection table

| Variables | *β* | *S.E* | *Z* | *P* | *OR (95%CI)* |
| --- | --- | --- | --- | --- | --- |
| FT4 | | | | | |
| **Water** |  |  |  |  |  |
| Centralized well water | 2.44 | 0.66 | 3.71 | **<0.001** | 11.49 (3.17 ~ 41.69) |
| Private well water | 3.35 | 1.05 | 3.17 | **0.002** | 28.38 (3.59 ~ 224.13) |
| **Kelp** |  |  |  |  |  |
| Consume occasionally | 0.79 | 0.38 | 2.09 | **0.036** | 2.20 (1.05 ~ 4.60) |
| **Nori** |  |  |  |  |  |
| Consume occasionally | 0.79 | 0.36 | 2.22 | **0.027** | 2.20 (1.10 ~ 4.42) |
| **Dairy products** |  |  |  |  |  |
| Consume occasionally | 1.18 | 0.56 | 2.10 | **0.036** | 3.26 (1.08 ~ 9.81) |
| **Radiation** |  |  |  |  |  |
| Yes | 0.87 | 0.34 | 2.58 | **0.010** | 2.39 (1.23 ~ 4.64) |
| **WI** | -0.01 | 0.00 | -2.45 | **0.014** | 0.99 (0.99 ~ 0.99) |
| TSH (low water iodine) | | | | | |
| **Smoke** |  |  |  |  |  |
| Current smoker | -2.18 | 1.07 | -2.05 | **0.041** | 0.11 (0.01 ~ 0.91) |
| **Water** |  |  |  |  |  |
| Centralized well water | 1.63 | 0.69 | 2.35 | **0.019** | 5.09 (1.31 ~ 19.84) |
| Private well water | 3.01 | 1.06 | 2.85 | **0.004** | 20.37 (2.56 ~ 162.17) |
| Bottled water | 1.14 | 0.54 | 2.09 | **0.036** | 3.11 (1.07 ~ 9.02) |
| **Nori** |  |  |  |  |  |
| Consume occasionally | 0.75 | 0.38 | 1.98 | **0.047** | 2.12 (1.01 ~ 4.47) |
| **UI** | 0.01 | 0.00 | 2.44 | **0.015** | 1.01 (1.01 ~ 1.01) |
| TSH (high water iodine) | | | | | |
| **Water** |  |  |  |  |  |
| Centralized well water | 0.88 | 0.29 | 3.01 | **0.003** | 2.42 (1.36 ~ 4.29) |
| Private well water | 3.20 | 1.04 | 3.09 | **0.002** | 24.64 (3.22 ~ 188.51) |
| Bottled water | 1.84 | 0.66 | 2.80 | **0.005** | 6.28 (1.74 ~ 22.68) |
| **Kelp** |  |  |  |  |  |
| Consume occasionally | 1.11 | 0.29 | 3.82 | **<0.001** | 3.04 (1.72 ~ 5.37) |
| **Nori** |  |  |  |  |  |
| Consume occasionally | 0.77 | 0.27 | 2.83 | **0.005** | 2.15 (1.27 ~ 3.66) |
| **Dairy products** |  |  |  |  |  |
| Consume occasionally | 1.21 | 0.39 | 3.07 | **0.002** | 3.35 (1.55 ~ 7.26) |
| Consume regularly | 1.32 | 0.41 | 3.23 | **0.001** | 3.73 (1.68 ~ 8.28) |
| **Fish** |  |  |  |  |  |
| Consume occasionally | 1.04 | 0.30 | 3.42 | **<0.001** | 2.82 (1.56 ~ 5.10) |
| **Types of edible oil** |  |  |  |  |  |
| Others | -0.79 | 0.39 | -2.05 | **0.041** | 0.45 (0.21 ~ 0.97) |
| **History of Neck Radiation** |  |  |  |  |  |
| Yes | 0.81 | 0.24 | 3.35 | **<0.001** | 2.24 (1.40 ~ 3.60) |
| **WI** | -0.01 | 0.00 | -4.10 | **<0.001** | 0.99 (0.99 ~ 0.99) |
| TPOAb(low water iodine) | | | | | |
| **Water** |  |  |  |  |  |
| Centralized well water | 3.37 | 1.05 | 3.21 | **0.001** | 29.02 (3.72 ~ 226.42) |
| **Egg** |  |  |  |  |  |
| Consume occasionally | 1.58 | 0.75 | 2.11 | **0.035** | 4.87 (1.12 ~ 21.20) |
| **Dairy products** |  |  |  |  |  |
| Consume regularly | 1.46 | 0.54 | 2.68 | **0.007** | 4.31 (1.48 ~ 12.51) |
| **Egg** |  |  |  |  |  |
| Consume occasionally | 1.34 | 0.38 | 3.55 | **<0.001** | 3.81 (1.82 ~ 7.97) |
| **Radiation** |  |  |  |  |  |
| Yes | 0.85 | 0.34 | 2.52 | **0.012** | 2.33 (1.21 ~ 4.50) |
| **UI** | 0.01 | 0.00 | 3.63 | **<0.001** | 1.01 (1.01 ~ 1.01) |
| **Height** | -0.07 | 0.02 | -2.76 | **0.006** | 0.93 (0.89 ~ 0.98) |
| TPOAb(high water iodine) | | | | | |
| **Water** |  |  |  |  |  |
| Centralized well water | 0.79 | 0.39 | 2.01 | **0.044** | 2.21 (1.02 ~ 4.79) |
| Private well water | 2.27 | 1.07 | 2.11 | **0.035** | 9.65 (1.18 ~ 79.06) |
| **Kelp** |  |  |  |  |  |
| Consume occasionally | 0.96 | 0.37 | 2.61 | **0.009** | 2.62 (1.27 ~ 5.38) |
| **Dairy products** |  |  |  |  |  |
| Consume regularly | 0.95 | 0.41 | 2.28 | **0.022** | 2.58 (1.14 ~ 5.81) |
| **Radiation** |  |  |  |  |  |
| Yes | 1.16 | 0.30 | 3.89 | **<0.001** | 3.18 (1.78 ~ 5.70) |
| **WI** | -0.01 | 0.00 | -3.37 | **<0.001** | 0.99 (0.99 ~ 0.99) |
| **Height** | -0.06 | 0.02 | -2.82 | **0.005** | 0.94 (0.90 ~ 0.98) |
| **Weight** | -0.03 | 0.01 | -2.02 | **0.043** | 0.97 (0.95 ~ 0.99) |
| TGAb (low water iodine) | | | | | |
| **Water** |  |  |  |  |  |
| Centralized well water | 2.00 | 0.79 | 2.51 | **0.012** | 7.36 (1.55 ~ 34.94) |
| Private well water | 3.03 | 1.05 | 2.88 | **0.004** | 20.60 (2.63 ~ 161.42) |
| **Kelp** |  |  |  |  |  |
| Consume occasionally | 1.03 | 0.38 | 2.71 | **0.007** | 2.81 (1.33 ~ 5.91) |
| **Egg** |  |  |  |  |  |
| Consume occasionally | 1.68 | 0.62 | 2.70 | **0.007** | 5.38 (1.59 ~ 18.26) |
| **Dairy products** |  |  |  |  |  |
| Consume occasionally | 1.15 | 0.44 | 2.61 | **0.009** | 3.17 (1.33 ~ 7.54) |
| Consume regularly | 2.01 | 0.66 | 3.07 | **0.002** | 7.50 (2.07 ~ 27.20) |
| **Fish** |  |  |  |  |  |
| Consume occasionally | 0.85 | 0.37 | 2.26 | **0.024** | 2.33 (1.12 ~ 4.85) |
| **UI** | 0.01 | 0.00 | 3.75 | **<0.001** | 1.01 (1.01 ~ 1.01) |
| TGAb (High water iodine) | | | | | |
| **Water** |  |  |  |  |  |
| Centralized well water | 1.04 | 0.35 | 2.97 | **0.003** | 2.84 (1.43 ~ 5.66) |
| **Kelp** |  |  |  |  |  |
| Consume occasionally | 1.04 | 0.35 | 2.97 | **0.003** | 2.84 (1.43 ~ 5.66) |
| **Nori** |  |  |  |  |  |
| Consume occasionally | 1.03 | 0.35 | 2.97 | **0.003** | 2.81 (1.42 ~ 5.55) |
| **Dairy products** |  |  |  |  |  |
| Consume occasionally | 1.20 | 0.44 | 2.74 | **0.006** | 3.33 (1.41 ~ 7.84) |
| Consume regularly | 1.44 | 0.49 | 2.97 | **0.003** | 4.23 (1.63 ~ 10.96) |
| **Radiation** |  |  |  |  |  |
| Yes | 0.91 | 0.27 | 3.40 | **<0.001** | 2.49 (1.47 ~ 4.20) |
| **WI** | -0.01 | 0.00 | -3.79 | **<0.001** | 0.99 (0.99 ~ 0.99) |
| Thyroid volume (low water iodine) | | | | | |
| **Gender** |  |  |  |  |  |
| Female | -0.78 | 0.23 | -3.39 | **<0.001** | -0.78 (-1.23 ~ -0.33) |
| **Smoke** |  |  |  |  |  |
| Current smoker | 0.89 | 0.28 | 3.21 | **0.001** | 0.89 (0.35 ~ 1.43) |
| Former smoker | 1.99 | 0.80 | 2.49 | **0.013** | 1.99 (0.42 ~ 3.55) |
| **Alcohol** |  |  |  |  |  |
| Yes | 0.49 | 0.24 | 2.07 | **0.039** | 0.49 (0.03 ~ 0.95) |
| **Tea** |  |  |  |  |  |
| Drink occasionally | 0.82 | 0.27 | 3.02 | **0.003** | 0.82 (0.29 ~ 1.34) |
| **Water** |  |  |  |  |  |
| Private well water | 0.78 | 0.26 | 3.01 | **0.003** | 0.78 (0.27 ~ 1.29) |
| **Eating habits** |  |  |  |  |  |
| Predominantly vegetarian | 1.81 | 0.87 | 2.09 | **0.037** | 1.81 (0.11 ~ 3.50) |
| Predominantly meat-based | 1.98 | 0.89 | 2.24 | **0.025** | 1.98 (0.25 ~ 3.72) |
| **Kelp** |  |  |  |  |  |
| Consume occasionally | 0.58 | 0.22 | 2.62 | **0.009** | 0.58 (0.15 ~ 1.01) |
| Consume regularly | 1.85 | 0.49 | 3.79 | **<0.001** | 1.85 (0.90 ~ 2.81) |
| **Nori** |  |  |  |  |  |
| Consume occasionally | 0.90 | 0.22 | 4.08 | **<0.001** | 0.90 (0.47 ~ 1.34) |
| Consume regularly | 1.16 | 0.44 | 2.63 | **0.009** | 1.16 (0.29 ~ 2.02) |
| **Dairy products** |  |  |  |  |  |
| Consume regularly | 1.16 | 0.26 | 4.45 | **<0.001** | 1.16 (0.65 ~ 1.67) |
| **Fish** |  |  |  |  |  |
| Consume occasionally | 0.68 | 0.22 | 3.10 | **0.002** | 0.68 (0.25 ~ 1.12) |
| Consume regularly | 1.26 | 0.60 | 2.10 | **0.036** | 1.26 (0.08 ~ 2.44) |
| **Radiation** |  |  |  |  |  |
| Yes | -0.90 | 0.23 | -4.01 | **<0.001** | -0.90 (-1.35 ~ -0.46) |
| **Height** | 0.03 | 0.01 | 2.30 | **0.021** | 0.03 (0.01 ~ 0.06) |
| **Weight** | 0.02 | 0.01 | 2.28 | **0.023** | 0.02 (0.01 ~ 0.04) |
| Thyroid volume (high water iodine) | | | | | |
| **Smoke** |  |  |  |  |  |
| Current smoker | 0.72 | 0.26 | 2.76 | **0.006** | 0.72 (0.21 ~ 1.24) |
| **Alcohol** |  |  |  |  |  |
| Yes | 0.48 | 0.22 | 2.17 | **0.031** | 0.48 (0.05 ~ 0.91) |
| **Water** |  |  |  |  |  |
| Centralized well water | 0.89 | 0.24 | 3.78 | **<0.001** | 0.89 (0.43 ~ 1.36) |
| Private well water | 0.64 | 0.31 | 2.08 | **0.038** | 0.64 (0.04 ~ 1.25) |
| **Saltiness Preference** |  |  |  |  |  |
| Bland | -0.62 | 0.24 | -2.59 | **0.010** | -0.62 (-1.09 ~ -0.15) |
| **Height** | 0.04 | 0.01 | 2.99 | **0.003** | 0.04 (0.01 ~ 0.06) |
| **Weight** | 0.03 | 0.01 | 3.90 | **<0.001** | 0.03 (0.02 ~ 0.05) |
| **BMI** | 0.06 | 0.02 | 2.58 | **0.010** | 0.06 (0.01 ~ 0.11) |

Stable 3 Table of variable assignment

| Variables | Assignment |
| --- | --- |
| **Gender** | Male=“0”; Female=“1” |
| **Smoke** | Never smoker=“0”; Current smoker=“1”; Former smoker=“2”; |
| **Alcohol** | No=“0”; Yes=“1”; |
| **Tea** | Rarely=“0”; Drink occasionally=“1”;Drink often=“2”; |
| **Kelp** | Rarely=“0”; Consume occasionally=“1”; Consume regularly=“2”; |
| **Nori** | Rarely=“0”; Consume occasionally=“1”; Consume regularly=“2”; |
| **Egg** | Rarely=“0”; Consume occasionally=“1”; Consume regularly=“2”; |
| **Dairy products** | Rarely=“0”; Consume occasionally=“1”; Consume regularly=“2”; |
| **Fish** | Rarely=“0”; Consume occasionally=“1”; Consume regularly=“2”; |
| **Water** | Tap water=“0”; Centralized well water=“1” Private well water=“2”; Bottled water=“3”; |
| **Eating habits** | Strictly vegetarian=“0”; Predominantly vegetarian=“1”; Predominantly meat-based=“2”; Strictly meat-based=“3”; |
| **Saltiness Preference** | Bland=“0”; Moderate=“1”; Salty=“2”; |
| **Types of edible oil** | Vegetable oil=“0”; Animal fat=“1”; Both=“2”; Others=“3”; |
| **Dietary Supplements** | None=“0”; Minerals=“1”; Multivitamins=“2”; Others=“3”; |
| **Tumor Conditions** | No=“0”; Yes=“1”; |
| **Cardiovascular Disease** | No=“0”; Yes=“1”; |
| **History of Neck Radiation** | No=“0”; Yes=“1”; |
| **Timing of Salt Addition** | At the start of cooking=“0”; During cooking=“1”; After cooking=“2”;Randomly added=“3” |

Stable 4 Definitions and data collection methods for all study variables.

| **Variable Category** | **Variable Name** | **Measurement Method / Data Source** | **Definition / Classification Criteria** |
| --- | --- | --- | --- |
| **Demographic Characteristics** | Age, Gender | Standardized Questionnaire | Verified via ID card or confirmed by self-report. |
|  | Height | Calibrated Stadiometer | Measured without shoes in an upright position, precise to 0.1 cm. |
|  | Weight | Calibrated Electronic Scale | Measured while fasting and wearing light clothing, precise to 0.1 kg. |
|  | BMI (Body Mass Index) | Calculated Index | Weight (kg)/Height ² (m²) |
| **Iodine Nutritional Indicators** | Urinary Iodine (UI) | Arsenic-Cerium Catalytic Spectrophotometry | Morning fasting urine or spot urine samples collected. Detected following the National Standard (WS/T 107-2006). |
|  | Water Iodine (WI) | Arsenic-Cerium Catalytic Spectrophotometry | Samples collected from the main household drinking water source. Detected following the National Standard (GB/T 5750.5-2006). |
| **Thyroid Function** | FT3, FT4, TSH | Automated Chemiluminescence Immunoassay Analyzer | Fasting venous blood samples collected and tested using standardized commercial kits. |
|  | TPOAb, TgAb | Automated Chemiluminescence Immunoassay Analyzer | Defined as Positive or Negative based on the reference ranges of the reagent kits. |
| **Health and Disease History** | Cardiovascular Disease, Tumor Conditions | Questionnaire | Defined as Yes (Physician-diagnosed) or No. |
|  | History of Neck Radiation | Questionnaire | Recent neck radiographic examination. |
| **Lifestyle Factors** | Smoke | Questionnaire | Classified into three categories: Never smoker, Current smoker, Former smoker |
|  | Alcohol | Questionnaire | Classified into two categories: No, Yes. |
|  | Tea | Questionnaire | Classified into three categories:Rarely, Drink occasionally, Drink often. |
| **Dietary Habits** | Water | Questionnaire | Classified into four categories: Tap water, Centralized well water, Private well water, Bottled water. |
|  | Fish, Nori, Kelp, Egg, Dairy products | Questionnaire | Classified into three categories based on frequency: Rarely, Consume occasionally, Consume regularly. |
|  | Eating habits | Questionnaire | Classified into four categories: Strictly vegetarian, Predominantly vegetarian, Predominantly meat-based, Strictly meat-based. |
|  | Saltiness Preference | Questionnaire | Classified into three categories: Bland, Moderate, Salty. |
|  | Timing of Salt Addition | Questionnaire | Classified as: At the start of cooking, During cooking, After cooking, Randomly added |
|  | Types of edible oil | Questionnaire | Classified as: Vegetable oil, Animal fat, Both, Others. |
|  | Dietary Supplements | Questionnaire | Classified as: None, Minerals, Multivitamins, Others. |


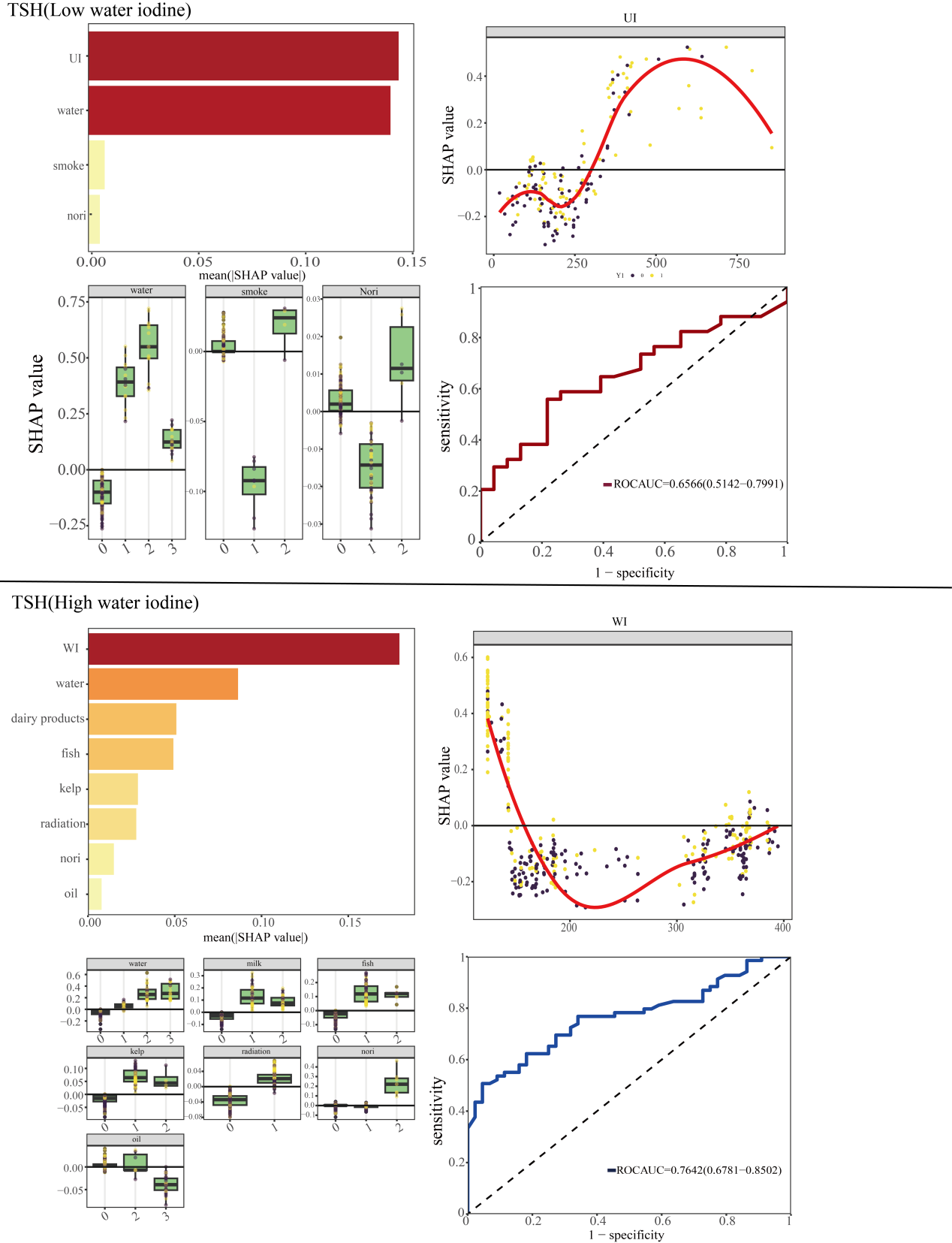


**Sfigure 1 Variable Importance Distribution Plot of TSH in Low and High Water Iodine Groups**


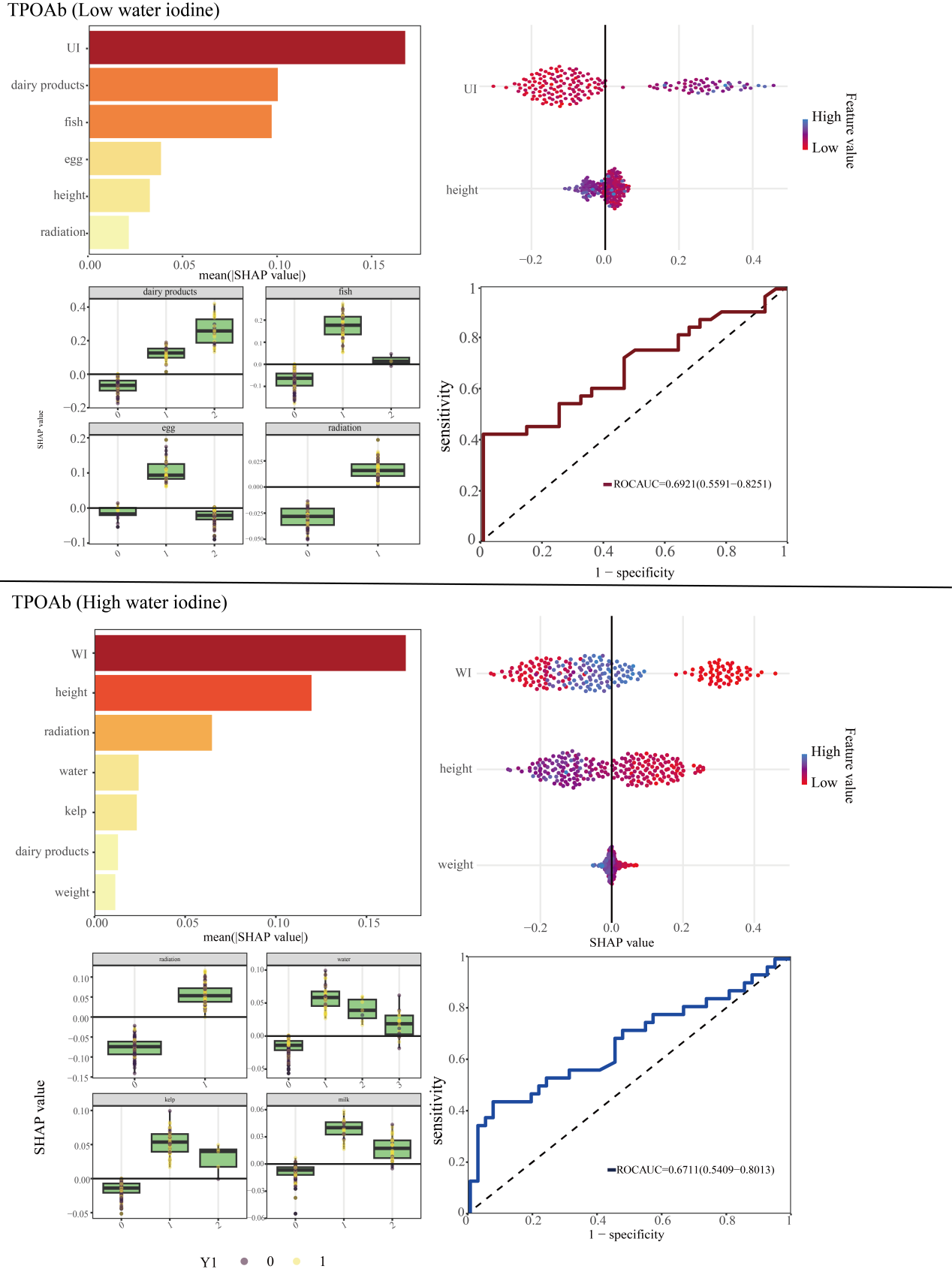


**Sfigure 2 Variable Importance Distribution Plot of TPOAb in Low and High Water Iodine Groups**

**
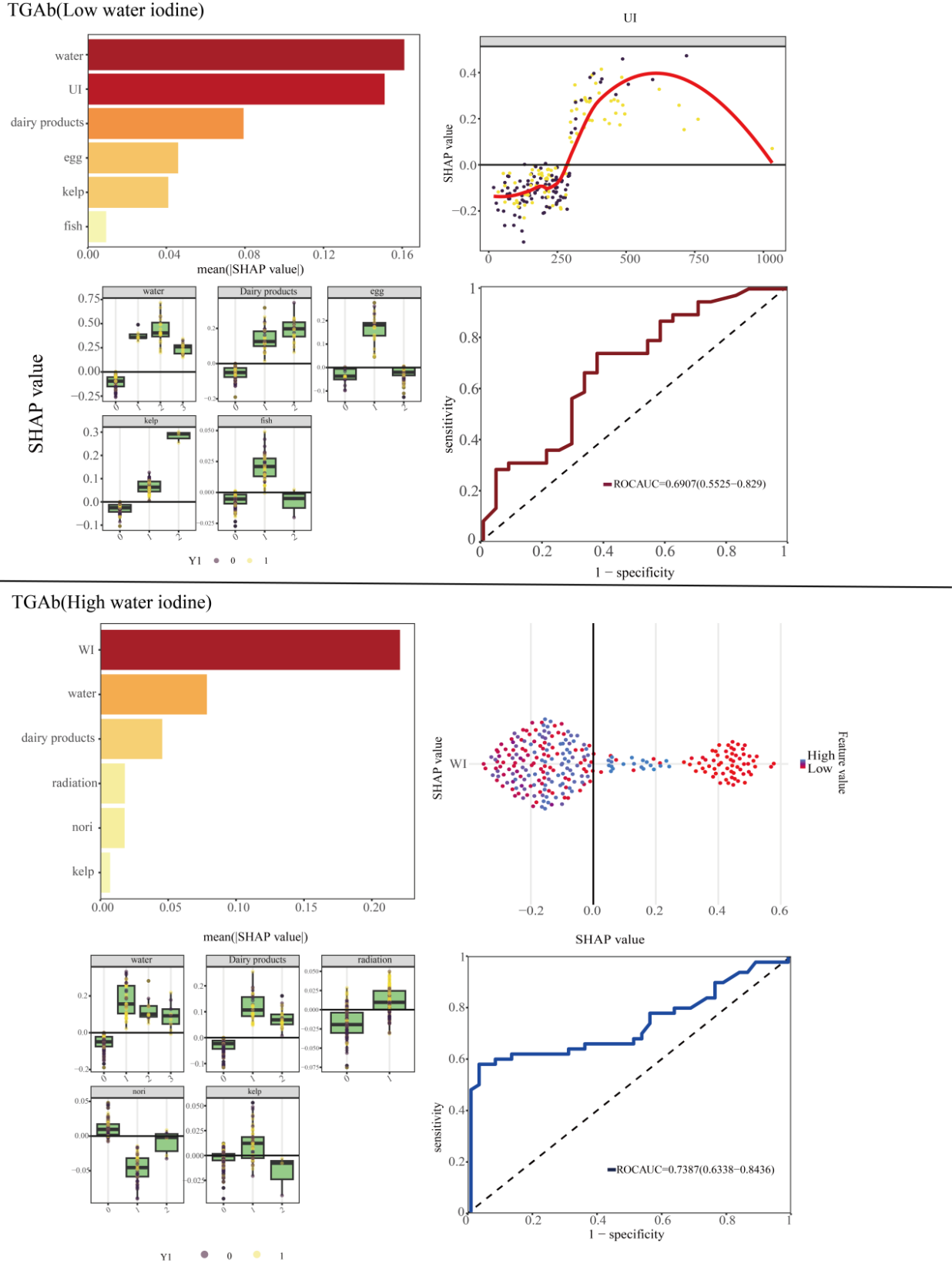
**

**Sfigure 3 Variable Importance Distribution Plot of TGAb in Low and High Water Iodine Groups**
